# Achieving Secondary Prevention Low-Density Lipoprotein Particle Concentration Goals Using Lipoprotein Cholesterol-Based Data

**DOI:** 10.1371/journal.pone.0033692

**Published:** 2012-03-29

**Authors:** Simon C. Mathews, Jaya Mallidi, Krishnaji Kulkarni, Peter P. Toth, Steven R. Jones

**Affiliations:** 1 Department of Medicine, The Johns Hopkins University, Baltimore, Maryland, United States of America; 2 Department of Epidemiology, The Johns Hopkins University School of Public Health, Baltimore, Maryland, United States of America; 3 Department of Internal Medicine, Baystate Medical Center/Tufts University, Springfield, Massachusetts, United States of America; 4 Research and Development, Atherotech Diagnostics Lab, Birmingham, Alabama, United States of America; 5 CGH Medical Center, Sterling, Illinois, and University of Illinois School of Medicine, Peoria, Illinois, United States of America; 6 Department of Medicine, Division of Cardiology, The Johns Hopkins University, Baltimore, Maryland, United States of America; John Hopkins Bloomberg School of Public Health, United States of America

## Abstract

**Background:**

Epidemiologic studies suggest that LDL particle concentration (LDL-P) may remain elevated at guideline recommended LDL cholesterol goals, representing a source of residual risk. We examined the following seven separate lipid parameters in achieving the LDL-P goal of <1000 nmol/L goal for very high risk secondary prevention: total cholesterol to HDL cholesterol ratio, TC/HDL, <3; a composite of ATP-III very high risk targets, LDL-C<70 mg/dL, non-HDL-C<100 mg/dL and TG<150 mg/dL; a composite of standard secondary risk targets, LDL-C<100, non-HDL-C<130, TG<150; LDL phenotype; HDL-C≥40; TG<150; and TG/HDL-C<3.

**Methods:**

We measured ApoB, ApoAI, ultracentrifugation lipoprotein cholesterol and NMR lipoprotein particle concentration in 148 unselected primary and secondary prevention patients.

**Results:**

TC/HDL-C<3 effectively discriminated subjects by LDL-P goal (F = 84.1, p<10^−6^). The ATP-III very high risk composite target (LDL-C<70, nonHDL-C<100, TG<150) was also effective (F = 42.8, p<10^−5^). However, the standard secondary prevention composite (LDL-C<100, non-HDL-C<130, TG<150) was also effective but yielded higher LDL-P than the very high risk composite (F = 42.0, p<10^−5^) with upper 95% confidence interval of LDL-P less than 1000 nmol/L. TG<150 and TG/HDL-C<3 cutpoints both significantly discriminated subjects but the LDL-P upper 95% confidence intervals fell above goal of 1000 nmol/L (F = 15.8, p = 0.0001 and F = 9.7, p = 0.002 respectively). LDL density phenotype neared significance (F = 2.85, p = 0.094) and the HDL-C cutpoint of 40 mg/dL did not discriminate (F = 0.53, p = 0.47) alone or add discriminatory power to ATP-III targets.

**Conclusions:**

A simple composite of ATP-III very high risk lipoprotein cholesterol based treatment targets or TC/HDL-C ratio <3 most effectively identified subjects meeting the secondary prevention target level of LDL-P<1000 nmol/L, providing a potential alternative to advanced lipid testing in many clinical circumstances.

## Introduction

Treatment based on low density lipoprotein cholesterol (LDL-C) levels has been the standard of care in treating patients with cardiovascular disease and those at risk. Data from multiple trials and studies, as summarized in the National Cholesterol Education Program Expert Panel on Detection, Evaluation, and Treatment of High Blood Cholesterol in Adults (NCEP ATP-III) have demonstrated the link between LDL-C levels and cardiovascular events as well as the corresponding impact of reducing LDL-C to reduce cardiovascular risk [1]. However, more recently atherogenic lipoprotein particle concentration measures such as low density lipoprotein particle concentration (LDL-P) determined by nuclear magnetic resonance (NMR) spectroscopy have outperformed LDL-C levels for prediction of vascular events in several studies [2]–[8]. Additional support for measurement of atherogenic lipoprotein particle burden has also come from consensus statements outlining the value of apolipoprotein B (apoB) as an alternative to LDL-P in identifying residual risk [9]–[11]. The superior performance of ApoB and LDL-P is understandable given the limited ability of LDL-C to predict corresponding particle concentration due to the heterogeneity of LDL particle size and density, particularly in the setting of insulin resistance. This potential limitation of LDL-C as a target of treatment is the basis of interest in the use of advanced lipid testing modalities, particularly in those at very high risk.

An update to the NCEP ATP-III guidelines established LDL-C<70 mg/dL as the primary treatment target in patients with very high risk, with secondary goals of non-HDL-C<100 mg/dL and optimum TG<150 mg/dL [12]. Patients at very high risk are defined as having the presence of established coronary artery disease or equivalent secondary prevention level risk diagnosis plus (1) multiple major risk factors (especially diabetes), (2) severe and poorly controlled risk factors (especially continued cigarette smoking), (3) multiple risk factors of the metabolic syndrome (especially high triglycerides ≥200 mg/dL plus non-HDL-C ≥130 mg/dL with low HDL-C [<40 mg/dL]), and (4) patients with acute coronary syndromes.^12^ In this context, we hypothesized that broader consideration of LDL-C, non-HDL-C, triglycerides (TG), and possibly the inclusion of LDL density, HDL-C, TG/HDL-C or TC/HDL-C may reliably allow simple lipid and lipoprotein cholesterol based targets to guide treatment to achieve the population equivalent prevention cutpoint of LDL-P<1000 nmol/L [13]. More specifically, we examined the relationship and performance of the following seven discriminating measures: TC/HDL<3, a composite of ATP-III very high risk targets (LDL-C<70 mg/dL, non-HDL-C<100 mg/dL and TG<150 mg/dL), a composite of standard secondary targets (LDL-C<100, non-HDL-C<130, TG<150), LDL phenotype A vs. A/B or B, HDL-C≥40, TG<150, and TG/HDL-C<3 in reaching an LDL-P goal of <1000 nmol/L. TG<150 mg/dL was used as a cutpoint per current ATP-III guidelines and is consistent with values used in the composites. Whereas the TG/HDL-C<3 cutpoint was tested due to its association with insulin resistance in overweight individuals [14] and density of LDL particles in type II diabetes [15].

## Methods

Informed consent was obtained from 148 patients located in southeastern Virginia and northeastern North Carolina, serially enrolled in an independent IRB approved protocol from a referral cardiology/lipidology patient population. Exclusion criteria included age <18 years and inability to provide informed consent. Venous blood was obtained by conventional phlebotomy and specimens were split for concurrent measurement of lipids and lipoproteins of interest.

Cholesterol concentration of major lipoprotein classes and subclasses was measured using the Vertical Auto Profile (VAP) procedure (Atherotech, Birmingham, AL). The VAP procedure has been described in detail previously [16], [17]. Briefly, the procedure consists of three major steps. In the first step, lipoprotein classes and subclasses are separated using a single vertical spin density gradient ultracentrifugation. A two-layer density gradient is prepared with 1.006 g/mL saline solution followed the sample aliquot of serum or plasma which has been diluted 40-fold with 1.21 g/mL KBr solution, submitted to ultracentrifugation at 65,000 rpm for 45 minutes. In the second step, the separated lipoprotein fractions are eluted and mixed with colorimetric reagents allowing spectrophotometric quatitation of cholesterol concentration by means of a proprietary continuous flow analyzer resulting in lipoprotein cholesterol concentration along the density gradient. In the third step, cholesterol concentration of each major lipoprotein class and subclass is determined by deconvolution and numerical integration of the digitized absorbance vs. elution time curve providing cholesterol concentrations of HDL, LDL, VLDL, Lp(a), IDL, and various subclasses of HDL, LDL and VLDL.

**Figure 1:**
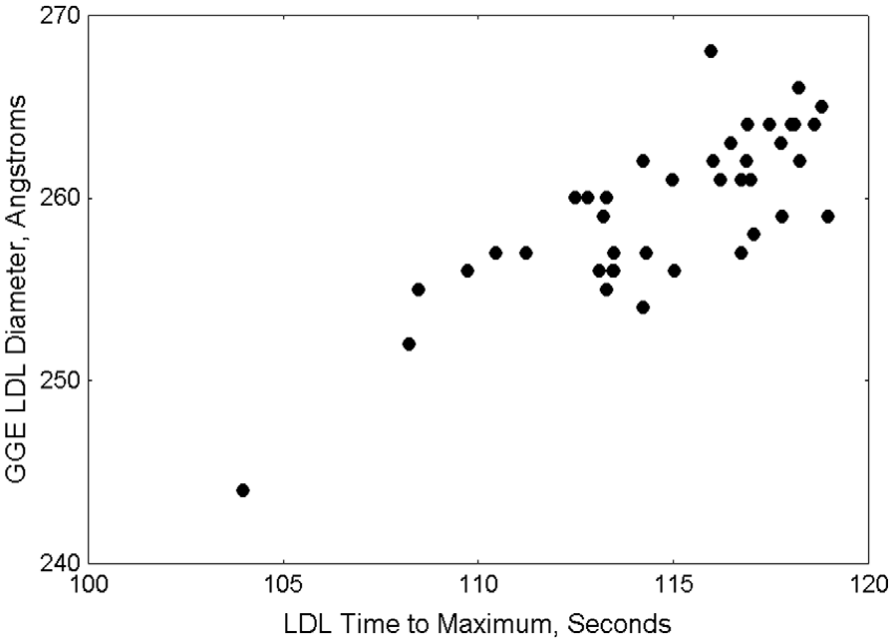
Typical relationship of average LDL particle size by gradient gel electrophoresis vs. VAP LDL peak maximum time density parameter; Pearson correlation coefficient, r = 0.80, p<10^−6^.

The VAP procedure provides LDL modal size-density phenotype. The assignment of phenotype class is based upon the relative position of the LDL cholesterol peak maximum on the eluted absorbance curve. Total sample elution times are normalized to 200 seconds, with the normalized elution time approximately inversely proportional to density. The LDL peak elution time ranges to assign LDL phenotypes by VAP are derived by comparison of LDL peak elution time values to corresponding LDL particle size determined by 2–16% polyacrylamide gradient gel electrophoresis (GGE) (Pacific Biometrics Inc., Seattle, WA) on split specimens. An example comparison of VAP LDL peak maximum time with average LDL size by GGE using scatter plot is shown in Figure 1. LDL peak elution time ≤112 seconds correspond to LDL size of ≤255 A° is defining small dense LDL phenotype B. Peak elution times ≥116 seconds correspond to LDL size ≥261 A° defining large buoyant LDL phenotype A. Patients with LDL maximum time 112–116 seconds are considered as having intermediate LDL phenotype A/B.

The accuracy of the VAP procedure was initially validated and is regularly calibrated against results obtained from the standard beta quantification procedure (Core Laboratories for Clinical Studies at Washington University, St. Louis, MO) using split serum specimens. Typically, Pearson correlation coefficients for lipoprotein cholesterol between the VAP procedure and beta quantification are: total cholesterol, 0.99; HDL, 0.99; LDL, 0.98; VLDL, 0.98; IDL, 0.78; Lp(a), 0.77; HDL_2_, 0.94, and HDL_3_, 0.91. VAP results are highly reproducible with typical between-days coefficient of variation: total cholesterol, 2.0%; HDL cholesterol, 2.9%; LDL cholesterol, 2.1%; VLDL cholesterol, 2.8%; IDL cholesterol, 8.2%; Lp(a) cholesterol, 9.1%; HDL_2_ cholesterol, 9.2%, and HDL_3_ cholesterol, 2.5%.

Serum lipoprotein particle concentrations were measured using quantitative proton NMR spectroscopy [18]–[20] (LipoScience; Raleigh, NC). Apo B and Apo AI measurements were performed using Architect/C8000 instrument (Atherotech; Birmingham, AL) and reagents (9D93-21 for Apo B and 9D92-21 for Apo AI) by Abbott Laboratories were standardized using WHO-International Reference Materials (SP1-01 for Apo AI and SP3-08 for Apo B) by participating in Apolipoprotein AI and B Standardization Program by Northwest Lipid Metabolism and Diabetes Research Laboratories, University of Washington, Seattle, WA. Lp(a)-P was measured as isoform independent whole particle concentration using the Denka-Seiken assay (Denka-Seiken, Tokyo, Japan) using the Abbott Architect/C8000 (Atherotech; Birmingham, AL). Triglycerides were measured using standard enzymatic methods also on the Abbott Architect/C8000 (Atherotech; Birmingham, AL).

### Statistical Analysis

Standard parametric analysis of variance (One way ANOVA) was used to compare the performance of various lipid measures to discriminate subjects by LDL-P goals and also to compare the various lipid measures between LDL density phenotypes among those patients meeting the composite very high risk ATP-III targets. To identify significant interactions between individual lipid measures, composite ATP-III targets and LDL density phenotype, covariate adjustments were done using analysis of covariance (ANCOVA). Classification performance of the various criteria was assessed using the C-statistic calculated as the area-under-the-curve (AUC) from LDL-P receiver operating characteristic (ROC) curves. An optimum classification point was calculated as the value of LDL-P where sensitivity and specificity were concurrently optimized.

Variables were log transformed as needed to meet Gaussian distribution requirements for parametric statistical analysis. Statistical analyses were performed using STATA 10 statistical software (StataCorp, College Station, Texas), Statistica 9 (Statsoft, Tulsa, Oklahoma) or Medcalc 11.5 (Medcalc Software, Mariakerke, Belgium). Two-sided P values less than 0.05 were considered statistically significant.

## Results

The mean age of subjects included in the analysis was 62.5 years (SD = 13 years). The cohort was 63.5% male and 36.5% female;. The following clinically documented diagnoses were present: type II diabetes, 15%; coronary artery disease, 63%; peripheral arterial disease, 16%; and treated or untreated dyslipidemia in patients referred for evaluation and treatment of dyslipidemia, not yet prescribed treatment., 79%. Diagnoses were based on conventional clinical diagnosis by patient's treating physicians as abstracted from the patient's medical record. The patients were not submitted to additional confirmatory or prospective initial testing for diagnoses as part of the study. Lipid modifying drug therapy included: statin, 78%; omega-3 polyunsaturated acids, 52%; nicotinic acid, 37%; ezetimibe, 29%; fibrate, 10%; bile acid sequestrant, 3%. Subjects frequently carried multiple diagnoses or were taking more than one lipid modifying drug.

We examined and compared the performance of a broad array of lipid measures, including a composite target consisting of the ATP-III very high risk secondary prevention goals (LDL-C<70 mg/dL, non-HDL-C<100 mg/dL and TG<150 mg/dL), TC/HDL-C<3 vs. ≥3, a composite of standard secondary prevention goals (LDL-C<100, non-HDL-C<130, TG<150), HDL-C<40 mg/dL vs. ≥40 mg/dL, LDL modal density by ultracentrifugation grouped as phenotype A (large, buoyant) vs. A/B or B (intermediate or small dense) phenotypes, TG<150 vs. TG>150, and TG/HDL-C<3 vs. TG/HDL-C>3. These potential univariate discriminators of LDL-P were examined across the entire group of subjects. One way ANOVA results are shown in Table 1.

**Table 1:** Univariate Discrimination of LDL-P and Performance by Lipid Criteria.

Univariate Discriminator (n)	LDL-P, nmol/L (95% CI)	Performance
**TC/HDL-C**		
<3 (67)	841 (784–898)	F = 84.1, p<10^−6^
≥3 (81)	1362 (1272–1456)	
**Very High Risk Composite**		
Achieved (53)	845 (775–914)	F = 42.8, p<10^−5^
Not Achieved (95)	1276 (1189–1364)	
**LDL Density Phenotype**		
A (67)	1062 (955–1169)	F = 2.85, p = 0.094
A/B or B (81)	1182 (1088–1275)	
**Secondary Prevention Composite**		
Achieved (74)	924 (863–985)	F = 42.0, p<10^−5^
Not Achieved (74)	1331 (1222–1440)	
**HDL-C**		
≥40 mg/dL (40)	1085 (992–1178)	F = 0.53, p = 0.47
<40 mg/dL (108)	1143 (1053–1234)	
**TG**		
<150 mg/dL (95)	1027 (954–1101)	F = 15.8, p = 0.0001
≥150 mg/dL (53)	1307 (1172–1443)	
**TG/HDL-C**		
<3 (86)	1036 (1057–1198)	F = 9.7, p = 0.002
≥3 (62)	1254 (1132–1376)	

TC/HDL-C most effectively discriminated subjects by LDL-P goal: TC/HDL-C<3, LDL-P 841 (784–898) vs. >3, LDL-P 1364 (1272–1456) nmol/L; F = 84.1, p<10^−6^. Achievement of the composite target of very high risk secondary ATP-III goals (LDL-C<70, non-HDL-C<100, TG<150) was also effective: LDL-P 845 (775–914) vs. 1276 (1189–1364) nmol/L; F = 42.8, p<10^−5^. The standard secondary prevention composite (LDL-C<100, non-HDL-C<130, TG<150) resulted in somewhat higher mean LDL-P than the very high risk composite discriminating statistically significantly between the 2 groups, F = 42.0, p<10^−5^. TG<150 also effectively discriminated subjects: LDL-P 1027 (954–1101) vs. 1307 (1172–1443) but not to the LDL-P cutpoint of 1000 nmol/L; F = 15.8, p = 0.0001. Evaluation of TG/HDL-C<3 produced a similar result: 1036 (1057–1198) vs. 1254 (1132–1376); F = 9.7, p = 0.002 respectively. Modal LDL density phenotype yielded a nonsignificant trend: A, LDL-P 1062 (955–1169) vs. A/B or B, 1182 (1088–1275) nmol/L; F = 2.85, p = 0.094 independent of significant interaction with ATP-III classification (F = 0.05, p = 0.99). The HDL-C cutpoint of 40 mg/dL did not discriminate alone or add discriminatory power to ATP-III targets: ≥40 mg/dL, LDL-P 1085 (992–1178) vs. <40 mg/dL, 1143 (1053–1234) nmol/L (F = 0.53, p = 0.47).

Secondary consideration of TC/HDL-C did not show independent additional predictive power over the ATP-III very high risk composite target with an expected significant interaction between the two classification criteria (F = 3.48, p = 0.03). LDL modal density phenotype showed a statistically significant trend toward improvement in discrimination of LDL-P target (F = 4.47, p = 0.04) and was independent, without interaction with the ATP-III very high risk target (F = 0.05, p = 0.99).

**Figure 2:**
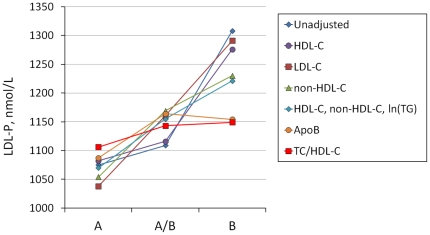
Mean LDL-P vs. LDL density phenotypes with covariate adjustments. Note that the effect of LDL modal density phenotype on LDL-P is rendered insignificant by HDL-C, ApoB and TC/HDL-C ratio.

Further analysis of adjusted LDL-P across LDL modal density phenotype A, A/B, and B is summarized in Table 2. LDL-P tends to increase with increasing LDL particle density class reaching near statistical significance (p = 0.052). Adjustment of mean LDL-P for ln(TG), ApoB, TC/HDL-C, ApoB/ApoAI, TC/HDL-C+ln(TG), and ApoB/ApoAI+ln(TG) reduced the effect of density phenotype on LDL-P below the level of statistical significance. However, when adjusted for age, HDL-C, ApoAI, LDL-C, non-HDL-C, or a composite HDL-C+non-HDL-C+ln(TG), the effect of density phenotype remained. Figure 2 summarizes LDL-P means across density phenotypes with and without covariate adjustment.

**Table 2:** LDL-P across LDL density phenotype adjusted for potentially confounding covariates.

Covariate	Phenotype A, (Mean, n = 67)	95% CI	Phenotype A/B (Mean, n = 56)	95% CI	Phenotype B (Mean, n = 25)	95% CI	*p*
Unadjusted	1075	972–1179	1109	995–1222	1308	1140–1475	0.052
age	1062	959–1165	1125	1012–1238	1307	1139–1476	0.062
HDL-C	1082	976–1189	1116	1002–1229	1276	1101–1450	0.18
ApoAI	1060	995–1165	1126	1012–1240	1310	1138–1481	0.057
ln(TG)	1106	1006–1025	1134	1028–1241	1171	1000–1242	0.81
LDL-C	1038	979–1097	1161	1097–1226	1291	1194–1388	0.00005
non-HDL-C	1054	994–1114	1169	1104–1234	1230	1133–1329	0.0034
HDL-C, non-HDL-C, ln(TG)	1070	1009–1130	1155	1091–1219	1221	1118–1324	0.036
ApoB	1087	1032–1141	1164	1104–1223	1154	1064–1245	0.14
TC/HDL-C	1106	1022–1191	1143	1051–1235	1149	1007–1292	0.80
ApoB/ApoAI	1117	1045–1190	1142	1063–1220	1122	1001–1244	0.89
TC/HDL-C, ln(TG)	1112	1026–1199	1143	1051–1236	1131	983–1280	0.89
ApoB/ApoAI, ln(TG)	1120	1046–1193	1142	1063–1222	1115	987–1242	0.89

**Figure 3:**
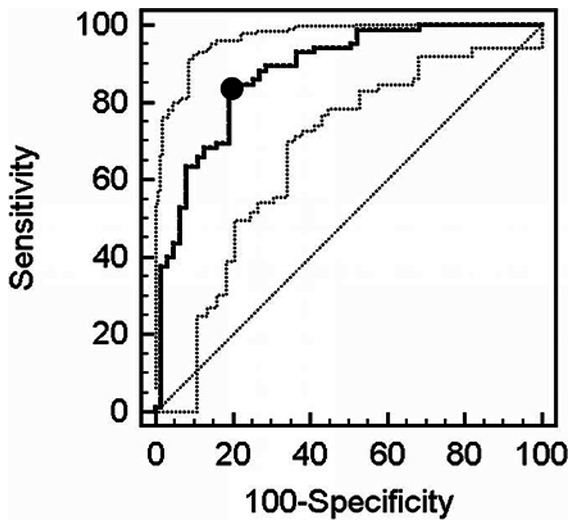
Receiver Operating Characteristic Curve analysis of TC/HDL-C for achieving LDL-P<1000 nmol/L target value (solid line) with 95% CI (broken lines). The diagonal broken line indicates the line of random chance or no discrimination. • Indicates optimized cutpoint for TC/HDL-C (2.96); Sensitivity 0.82; Specificity 0.81.

We determined optimized lipoprotein, lipoprotein cholesterol and lipid based parameters using ROC curves to predict achieving LDL-P<1000 nmol/L (Table 3). For TG, the optimum cutpoint was 99 mg/dL (AUC = 0.710 [0.628–0.782]); for LDL-C, the optimum cutpoint was 65 mg/dL (AUC = 0.864 [0.798–0.915]); for non-HDL-C, the optimum cutpoint was 90 mg/dL (AUC = 0.877 [0.813–0.925]); for HDL-C, the optimum cutpoint was 54 mg/dL (AUC = 0.596 [0.513–0.676]); for TC/HDL-C ratio, the optimum cutpoint was 2.96 (AUC = 0.877 [0.813–0.925], Figure 3); for ApoB, the optimum cutpoint was 70 mg/dL (AUC = 0.886 [0.822–0.933]); for ApoB/ApoAI ratio, the optimum cutpoint was 0.50 (AUC = 0.888 [0.826–0.934]).

**Table 3:** Receiver operating characteristic curve analysis of selected predictors of achieving LDL-P<1000 nmol/L target value.

Parameter	AUC (95% CI)	Optimum Cutpoint	Sensitivity	Specificity
Triglycerides, mg/dL	0.710 (0.628–0.782)	99	0.73	0.62
LDL-C, mg/dL	0.864 (0.798–0.915)	65	0.88	0.68
Non-HDL-C, mg/dL	0.877 (0.813–0.925)	90	0.79	0.79
HDL-C, mg/dL	0.596 (0.513–0.676)	54	0.81	0.43
TC/HDL-C ratio	0.877 (0.813–0.925)	2.96	0.82	0.81
ApoB, mg/dL	0.886 (0.822–0.933)	70	0.75	0.86
ApoB/ApoAI ratio	0.888 (0.826–0.934)	0.5	0.78	0.87

HDL-C results in classification significantly differ from chance, p = 0.049; all others, p<0.0001.

Separate analyses were performed to calculate the predicted optimal LDL-P value for classification using 2 composite criterion based classifications. The first criterion was the ATP-III very high risk composite of LDL-C, non-HDL-C, and TG targets. The second composite criterion was derived from the optimum lipoprotein cholesterol and lipid univariate values from ROC analysis for LDL-P<1000 nmol/L classification (LDL-C = 65 mg/dL, non-HDL-C = 90 mg/dL, and TG = 99 mg/dL) as summarized in table 4. ATP-III very high risk composite yielded an optimum cutpoint of 1107 nmol/L whereas the criterion based on ROC optimized composite LDL-C, non-HDL-C and TG predicted an optimal LDL-P cutpoint of 910 nmol/L.

**Table 4:** Receiver operating characteristic curve analysis of LDL-P as a predictor of achieving composite LDL-C, non-HDL-C, and triglyceride composite targets, ATP-III very high risk (ATP-III Composite) or composite of univariate ROC optimized LDL-P cutpoints, nmol/L, for the same parameters (table A, ROC Optimized).

Parameter	AUC (95% CI)	Optimum LDL-P Cutpoint	Sensitivity	Specificity
ATP-III composite	0.824 (0.752–0.883)	1107	0.86	0.62
ROC Optimized composite	0.886 (0.822–0.933)	910	0.82	0.83

Classifications significantly differ from chance, p<0.0001.

## Discussion

A simple composite of ATP-III very high risk lipoprotein cholesterol based treatment targets or TC/HDL-C ratio<3 effectively identified subjects meeting the secondary prevention target level of LDL-P<1000 nmol/L. The composite target is particularly useful given the widespread familiarity with its underlying parameters based on existing ATP-III lipid guidelines. Consequently, in a general unselected clinical cohort, simple lipoprotein cholesterol and triglyceride targets have the potential to identify patients achieving high risk LDL-P goals without advanced testing. These findings support the use of familiar, simple, widely available, and well-standardized parameters as risk metrics in evaluating very high risk secondary prevention patients.

Our study demonstrated that traditional cholesterol based measurements can be used effectively as a proxy for an LDL-P target. There is little contention in using LDL-C to identify patients at higher risk for cardiovascular disease. However, the metric's role as a treatment target and a measure of treatment success is a source of debate [19], [21]–[23]. This concern is fundamentally based on the pathophysiology of cardiovascular disease as it directly relates to a gradient driven process: LDL particle concentration dictates the flow of LDL particles into the arterial wall [24], leading to subsequent subendothelial retention of LDL and other ApoB containing lipoproteins which initiates the pathway of atherosclerotic disease [25]. This atherogenic particle concentration dependent process is often poorly reflected in LDL-C values and consequently results in underestimation of risk. This is highlighted by the considerable cardiovascular risk present even in aggressively statin-treated patients [1], [12], [26]–[28] and by the discordance in decline between LDL-C values and atherogenic particle concentration [23]. Additionally, the heterogeneity of LDL-P at a given LDL-C level is well-described [19], [22] as well as its impact on cardiovascular outcomes [2]. These collective findings support the notion of residual risk present at lower LDL-C levels and the subsequent use of alternative modalities such as LDL-P to identify it.

Our study demonstrated that traditional cholesterol based lipid measures can be used effectively as a proxy for LDL-P target. TC/HDL<3 and a composite target of LDL-C, non-HDL-C, and TG measures more accurately accounted for residual risk compared to LDL-C alone. Our findings support the growing evidence for using alternatives to the solely LDL-C based risk management framework. Of note, the TC/HDL-C ratio is currently used as an ostensible secondary treatment target in the Canadian Guidelines for Cholesterol Management [29], but is explicitly not recommended as a secondary target in ATPIII because it “will divert priority from specific lipoprotein fractions as targets of therapy” [1]. Our study supports further inclusion and discussion of this metric as new guidelines are established. While HDL-C did not show independent additional predictive power over the ATP-III composite target, LDL density phenotype type demonstrated a trend toward statistical significance when considered along with composite ATP-III very high risk targets but did not add predictive power to the TC/HDL-C<3 criterion or similar Apolipoprotein based criteria.

Unadjusted mean LDL-P increased across LDL density phenotypes however this effect was ameliorated by adjustment of the data for TC/HDL-C ratio, ApoB or ApoB/ApoAI ratio but not simple or composites of lipid or lipoprotein cholesterol covariates. These findings suggest that TC/HDL-C carries information reflecting LDL density and hence particle excess not found in simple lipid variables such as LDL-C and nonHDL-C. Of note, ApoB outperformed non-HDL-C in this regard, highlighting the limitation of non-HDL-C as a surrogate for ApoB despite the strong correlation between the two measures.

These results are consistent with broader evidence demonstrating the predictive power of density phenotype, particularly small, dense LDL particles' (phenotype B) association with higher risk of coronary heart disease [30]–[32]. As a result, the added discriminatory power of LDL density phenotype, particularly in the context of a very high risk composite ATP-III target, should be further investigated within a larger cohort and specifically compared head to head with a simple TC/HDL-C criterion.

Lastly, ROC curve optimization to predict LDL-P<1000 nmol/L using individual parameters identified cutpoints that were lower than current ATP-III very high risk targets: LDL-C<65 mg/dL, non-HDL-C<90 mg/dL, and TG<99 mg/dL. Additionally, a complementary analysis optimizing a composite of TG, LDL-C, and non-HDL-C suggests that more aggressive lipoprotein cholesterol and TG target values than currently recommended may be beneficial for those at highest risk to ensure adequate reduction in atherogenic particle burden.

### Limitations

Our study population was relatively small and composed of serially collected, heterogeneous, and multiply treated patients. These pilot study characteristics inherently limit the statistical power and generalizability of the analysis, particularly in detecting the contribution of LDL modal density phenotype. The heterogenous nature of the cohort is typical of clinical practice weighted heavily with patients with secondary prevention level risk. Accordingly, we focused our analysis on very high risk lipid targets and those patients at highest risk which limits the ability to generalize the results to lower risk or primary prevention patients. The targets and high risk profile emphasized in this study include the patient population most likely to undergo additional testing in clinical practice.

### Conclusion

Our study provides a potential alternative way of accounting for atherogenic particle burden and associated risk using a composite very high risk ATP-III target or TC/HDL-C ratio. Given the low cost, near universal availability, and broader clinician understanding of these options, they are arguably the preferred risk metrics for most patients. Ultimately, further research is needed to refine the indications for the incremental use of advanced measures of atherogenic lipoprotein particle burden after intensive composite lipid and lipoprotein cholesterol or TC/HDL-C targets have been met in high risk prevention.
